# Feasibility of a cryopreservation of cultured human corneal endothelial cells

**DOI:** 10.1371/journal.pone.0218431

**Published:** 2019-06-21

**Authors:** Naoki Okumura, Takato Kagami, Kyoko Watanabe, Saori Kadoya, Masakazu Sato, Noriko Koizumi

**Affiliations:** Department of Biomedical Engineering, Faculty of Life and Medical Sciences, Doshisha University, Kyotanabe, Japan; University of Florida, UNITED STATES

## Abstract

Transparency of the cornea is essential for vision and is maintained by the corneal endothelium. Consequently, corneal endothelial decompensation arising from irreversible damage to the corneal endothelium causes severe vision impairment. Until recently, transplantation of donor corneas was the only therapeutic choice for treatment of endothelial decompensation. In 2013, we initiated clinical research into cell-based therapy involving injection of a suspension of cultured human corneal endothelial cells (HCECs), in combination with Rho kinase inhibitor, into the anterior chamber. The aim of the present study was to establish a protocol for cryopreservation of HCECs to allow large-scale commercial manufacturing of these cells. This study focused on the effects of various cryopreservation reagents on HCEC viability. Screening of several commercially available cryopreservation reagents identified Bambanker hRM as an effective agent that maintained a cell viability of 89.4% after 14 days of cryopreservation, equivalent to the cell viability of 89.2% for non-cryopreserved control cells. The use of Bambanker hRM and HCECs at a similar grade to that used clinically for cell based therapy (passage 3–5 and a cell density higher than 2000 cells/mm^2^) gave a similar cell density for cryopreserved HCECs to that of non-preserved control HCECs after 28 days of cultivation (2099 cells/mm^2^ and 2111 cells/mm^2^, respectively). HCECs preserved using Bambanker hRM grew in a similar fashion to non-preserved control HCECs and formed a monolayer sheet-like structure. Cryopreservation of HCECs has multiple advantages including the ability to accumulate stocks of master cells, to transport HCEC stocks, and to manufacture HCECs on demand for use in cell-based treatment of endothelial decompensation.

## Introduction

The cornea is a transparent tissue that works as a “lens” within the eye to focus light onto the retina. Consequently, the cornea must retain its transparency if it is to serve this function. This transparency is maintained by the corneal endothelium, which regulates water flow between the aqueous humor and the corneal stroma by pump-and-leak barrier functions [1]. However, the corneal endothelial cells (CECs) that perform this function have severely limited proliferative capacity [2], so any severe damage to the corneal endothelium, such as that arising from pathological conditions like Fuchs endothelial corneal dystrophy or from iatrogenic damage during cataract surgery, causes irreversible cell loss. A reduction in the CEC density below a critical level (usually less than 500 cells/mm^2^) disrupts water regulation by the corneal endothelium and leads to the loss of corneal transparency [3].

At present, the only treatment for this corneal endothelial decompensation is transplantation of a donor cornea: no other treatment, including the use of pharmaceutical agents, is available [4]. The most common transplantation was originally full thickness penetrating keratoplasty, performed since the 1900s [4], but corneal endothelial transplantations, such as Descemet stripping automated endothelial keratoplasty (DSAEK) and Descemet membrane endothelial keratoplasty (DMEK), have gained popularity in the last decade [5–8]. However, tissue engineering technology is now receiving increased attention, as researchers view this as a way to overcome the main problems of corneal transplantations, which include a shortage of donor corneas, late graft failure due to continuous cell loss, graft rejection, and the learning curve involved in performing corneal transplant procedures [9–14].

In 2013, we initiated clinical research into cell-based therapy involving injection of a suspension of cultured human corneal endothelial cells (HCECs), in combination with a Rho kinase inhibitor, into the anterior chamber [15]. We recently reported the clinical outcome of the first 11 cases of human patients with endothelial decompensation who underwent this cell-based treatment. All 11 cases recovered corneal transparency and none experienced any severe adverse effects, either local or systemic [15]. For this clinical research, the HCECs were obtained from donor corneas and expanded in in vitro culture in the cell processing center (CPC) at the Kyoto Prefectural University of Medicine. The HCECs were harvested from a culture plate, placed in a tube in the form of a cell suspension, and immediately transported to the operating room in the same facility [15]. This clinical research showed the effectiveness and safety of this new procedure, so our next goal is to obtain approval for this cell-based therapy from regulatory authorities, including the Pharmaceuticals and Medical Devices Agency (PMDA), the Food and Drug Administration (FDA), and the European Medicines Agency (EMA). This approval will allow HCECs to be marketed as a product, thereby eventually allowing physicians and patients worldwide to access this new therapy. We are currently optimizing the entire protocol, from improving the efficiency of in vivo expansion to establishment of large-scale commercial cell culture protocols, transportation methods, quality control practices, and cryopreservation procedures to enable the CPC to manufacture and provide HCECs as a product [16–18].

The lack of efficient cryopreservation techniques is a current bottleneck in the manufacturing and marketing of HCECs. Establishment of an effective cryopreservation method for these cells will provide several advantages for cell-based therapy: 1) primary culture from donor corneas and in vitro expansion can be performed in a single CPC, 2) master cells can be stocked, 3) cells can be transported from the CPC to other CPCs as frozen stocks, 4) cells can be prepared from these stocks rather than being manufactured on demand for surgery. Therefore, multiple research groups have reported the feasibility of the cryopreservation of HCECs [19–23]. In the current study, we have screened several cryopreservation reagents for their effectiveness in preserving HCECs. We also evaluated the effect of cryopreservation on cell growth, cell density, and functional HCEC phenotypes.

## Materials and methods

### Ethics statement

Donor corneas were provided by SightLife^TM^ (Seattle, WA). Informed written consent for eye donation for research purposes was obtained from the next of kin of the deceased donors. Corneas were recovered under the tenets of the Uniform Anatomical Gift Act (UAGA) of the particular state. Human corneas were handled in accordance with the tenets set forth in the Declaration of Helsinki.

### Culture of corneal endothelial cells

Ten human donor corneas were used in the current study. All donors were >40 years of age. All corneas had been stored at 4°C in Optisol (Chiron Vision, Irvine, CA) for less than 14 days before use in experiments. Human corneal endothelial cells (HCECs) were cultured according to previously described protocols [24]. Briefly, Descemet’s membranes containing the corneal endothelium were stripped from the donor corneas and incubated with 1 mg/mL collagenase A (Roche Applied Science, Penzberg, Germany) at 37° C for 12 hours. The HCECs were recovered after washing with OptiMEM-I (Life Technologies Corp., Carlsbad, CA) three times and were seeded in one well of a 48-well plate coated with laminin E8 fragments (iMatrix-511; Nippi, Incorporated, Tokyo, Japan).

Culture medium was prepared by conditioning basal medium mix (OptiMEM-I, 8% fetal bovine serum [FBS], 5 ng/mL epidermal growth factor [EGF; Thermo Fisher Scientific], 20 μg/mL ascorbic acid [Sigma-Aldrich, St. Louis, MO], 200 mg/L calcium chloride, 0.08% chondroitin sulfate [Sigma-Aldrich], and 50 μg/mL gentamicin [Thermo Fisher Scientific]) with NIH-3T3 cells according to a previously reported protocol [24]. Briefly, NIH-3T3 cells were treated with 4 μg/mL mitomycin C (Kyowa Hakkko Kirin Co., Ltd., Tokyo, Japan) for 2 hours and then seeded onto a culture plate at a cell density of 2 × 10^4^ cells/cm^2^. The NIH-3T3 cells were cultured with fresh basal medium mix for 24 hours; this basal medium mix was then collected, filtered through a 0.22-μm filtration unit (EMD Millipore Corporation, Billerica, MA), and used to culture the corneal endothelial cells. Cultured HCECs at passages 5 through 10 and with cell density of 1000–1500 cells/mm^2^ were used for the screening experiments. HCECs at passages 3 through 5 and with cell density >2000 cells/mm^2^ were used as clinical grade cells to evaluate the effectiveness of the cryopreservation protocol. Cell density was evaluated using ImageJ software (National Institutes of Health, Bethesda, MD, USA).

### Cryopreservation of HCECs

The HCECs were washed in Ca^2+^- and Mg^2+^-free phosphate buffered saline (PBS), trypsinized with TrypLE Select Enzyme (10X) (Thermo Fisher Scientific) for 15 minutes at 37°C, and recovered from the culture plate in Corning Cryogenic Vials 2ml (Sigma-Aldrich). After counting cell numbers, HCECs were centrifuged at 280 G for three minutes. The supernatant was removed and cryopreservation reagent (1 ml for 5×10^5^ cells), which was maintained at 4°C, was added. Immediately after gently pipetting the HCECs into cryopreservation reagent in Corning Cryogenic Vials, the HCECs were frozen at -80°C in Bicell (Nihon Freezer Co., Ltd. Tokyo, Japan), which was maintained at -80°C before use. Bicell was designed to freeze cells in approximately three hours, according to the manufacture’s protocol. After freezing in Bicell for 24 hours, the Corning Cryogenic Vials containing HCECs were removed from the Bicell and stored in liquid nitrogen (-196°C) for an additional 13 days. After this cryopreservation period, the HCECs were incubated at 37°C in a water bath for 1–2 minutes, centrifuged at 190 G for five minutes, and resuspended in culture medium. Cell viability was determined by staining the dead cells with 0.5% Trypan Blue stain (Nacalai tesque, Kyoto, Japan) after resuspension in culture medium. The HCECs were then seeded in a culture plate for subsequent experiments.

The following cryopreservation reagents were used in the screening experiments: Cellbanker 2 (Nippon Genyaku Kogyou Co., Ltd, Ltd, Fukushima, Japan), Bambanker (Nippon Genetics Co., Ltd, Tokyo, Japan), KM Banker (Cosmo Bio Co., Ltd, Tokyo, Japan), Stem-Cellbanker (Nippon Genyaku Kogyou Co., Ltd), Bambanker hRM (Nippon Genetics Co., Ltd), and ReproCryo DMSO Free RM (ReproCELL Inc., Kanagawa, Japan). Opti-MEM + 10% v/v dimethyl sulfoxide (DMSO; Nacalai tesque) + 10% v/v FBS was used as a control cryopreservation reagent.

### Staining

HCECs cultured on a 48-well cell culture plate were fixed in 0.5% paraformaldehyde for 45 minutes, permeabilized with 1%Triton X-100 for 5 minutes, and incubated with 2% bovine serum albumin (BSA) for 30 minutes. The HCECs were incubated with the following primary antibodies for 45 minutes at 37°C: ZO-1 (1:200, Zymed Laboratories, South San Francisco, CA), N-cadherin (1: 300, BD Biosciences), and Na^+^/K^+^-ATPase (1:200, Merck Millipore). Either Alexa Fluor 488-conjugated goat anti-rabbit IgG (Thermo Fisher Scientific) or Alexa Fluor 594-conjugated goat anti-mouse IgG (Thermo Fisher Scientific) was diluted at 1:500 in BSA, and the samples were incubated in the secondary antibodies for 45 minutes at 37°C. Actin was stained by incubating samples with a 1:200 diluted Alexa Fluor 546-conjugated phalloidin (Life Technologies Corp.) for 45 minutes at 37°C. Nuclei were stained with 1:1000 diluted 4’,6-diamidino-2-phenylindole dihydrochloride (DAPI) (Vector Laboratories, Burlingame, CA). The samples were examined by fluorescence microscopy (BZ-9000; Keyence, Osaka, Japan).

### Statistical analysis

The statistical significance (*P*-value) for mean values in two-sample comparisons was determined with the Student’s t-test. The statistical significance of comparisons of multiple sample sets was determined with Dunnett’s multiple-comparisons test. Results were expressed as mean ± standard deviation. A *P* value less than 0.05 was considered statistically significant.

## Results

### Cryopreservation of HCECs by using Opti-MEM + 10%DMSO + 10%FBS

**Fig 1:**
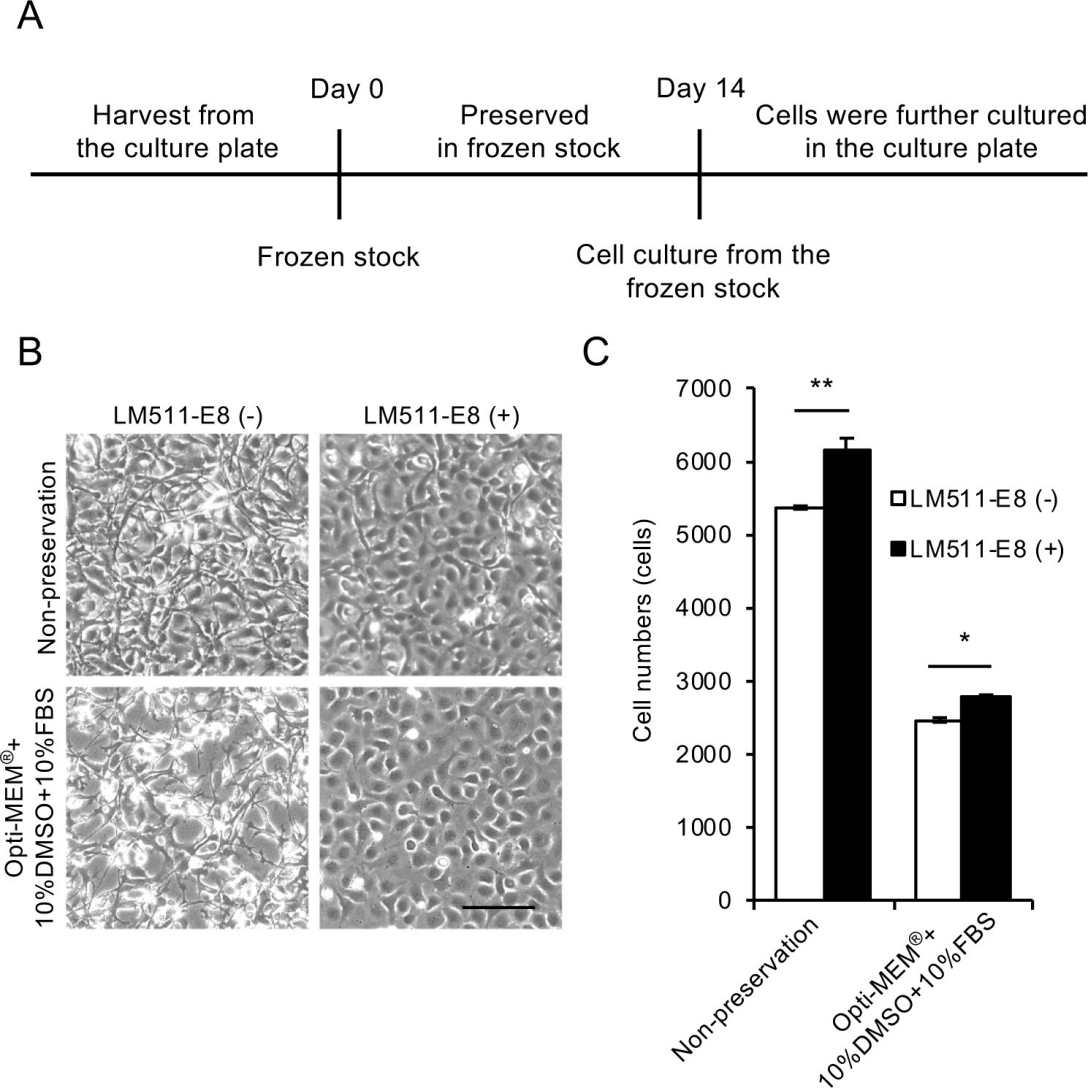
Cryopreservation of human corneal endothelial cells (HCECs) using Opti-MEM + 10%DMSO + 10%FBS. (A) Time schedule for HCEC cryopreservation is shown. HCECs were harvested from the culture plate and suspended in cryopreservation reagent. After cryopreservation for 14 days, HCECs were seeded on the culture plate and cultured. (B) HCECs were suspended in Opti-MEM + 10%DMSO + 10%FBS, cryopreserved for 14 days, and seeded at the cell density of 6.0×10^4^ in one well of a 48-well culture plate for 24 hours. Three phase contrast images were obtained from each well of the 48-well culture plate, and randomly selected images by masked examiner are shown. Phase contrast microscopy showed fewer cells adhering to the culture plate, presumably due to the death of HCECs during preservation (left, lower), when compared to HCECs cultured without prior cryopreservation, shown as a control (left, upper). Inclusion of a laminin-511 E8 fragment increased the numbers of adhered HCECs. Scale bar: 200 μm. (C) Both the non-preserved and cryopreserved HCECs showed significantly increased cell numbers in response to the laminin-511 E8 fragment coating after 24 hours of culture. However, the cell numbers for the cryopreserved HCECs approximately half that obtained with the non-preserved HCECs. Experiments were performed in triplicate (n = 3). **p<0.01, *p<0.05.

HCECs were cryopreserved for 14 days, as shown in Fig 1, and seeded at the cell density of 6.0×10^4^ in one well of a 48-well culture plate. We first examined the suitability of Opti-MEM + 10%DMSO + 10%FBS for cryopreservation, as mixture of basal medium with DMSO and FBS is often used as cryopreservation reagent for various cell types. Phase contrast images obtained 24 hours after culturing HCECs after preservation showed that fewer cells adhered to the plate, indicating larger numbers of presumably dead cells (Fig 1B, lower left) when compared to HCECs cultured without prior cryopreservation (Fig 1B, upper left). We previously reported that the E8 fragment of laminin-511, a component of the basement membrane of corneal endothelium (Descemet’s membrane), promoted HCEC culture [24], so we also evaluated the effect of this fragment on cell culture after cryopreservation. The phase contrast images showed that laminin-511 E8 fragment increased the numbers of adhering HCEC (Fig 1B). In terms of cell morphology, HCECs cultured without the laminin-511 E8 fragment exhibited fibroblastic morphology, while HCECs cultured with the laminin-511 E8 fragment exhibited less fibroblastic and more corneal endothelial cell-like morphology (Fig 1B). Cell numbers after 24 hours of cell culture were decreased by approximately one half when HCECs were cryopreserved in Opti-MEM + 10%DMSO + 10%FBS when compared to cultures of non-preserved HCECs, but culture with the laminin-511 E8 fragment significantly increased the cell numbers (Fig 1C).

### Screening of preservation reagents for cryopreservation of HCECs

**Fig 2:**
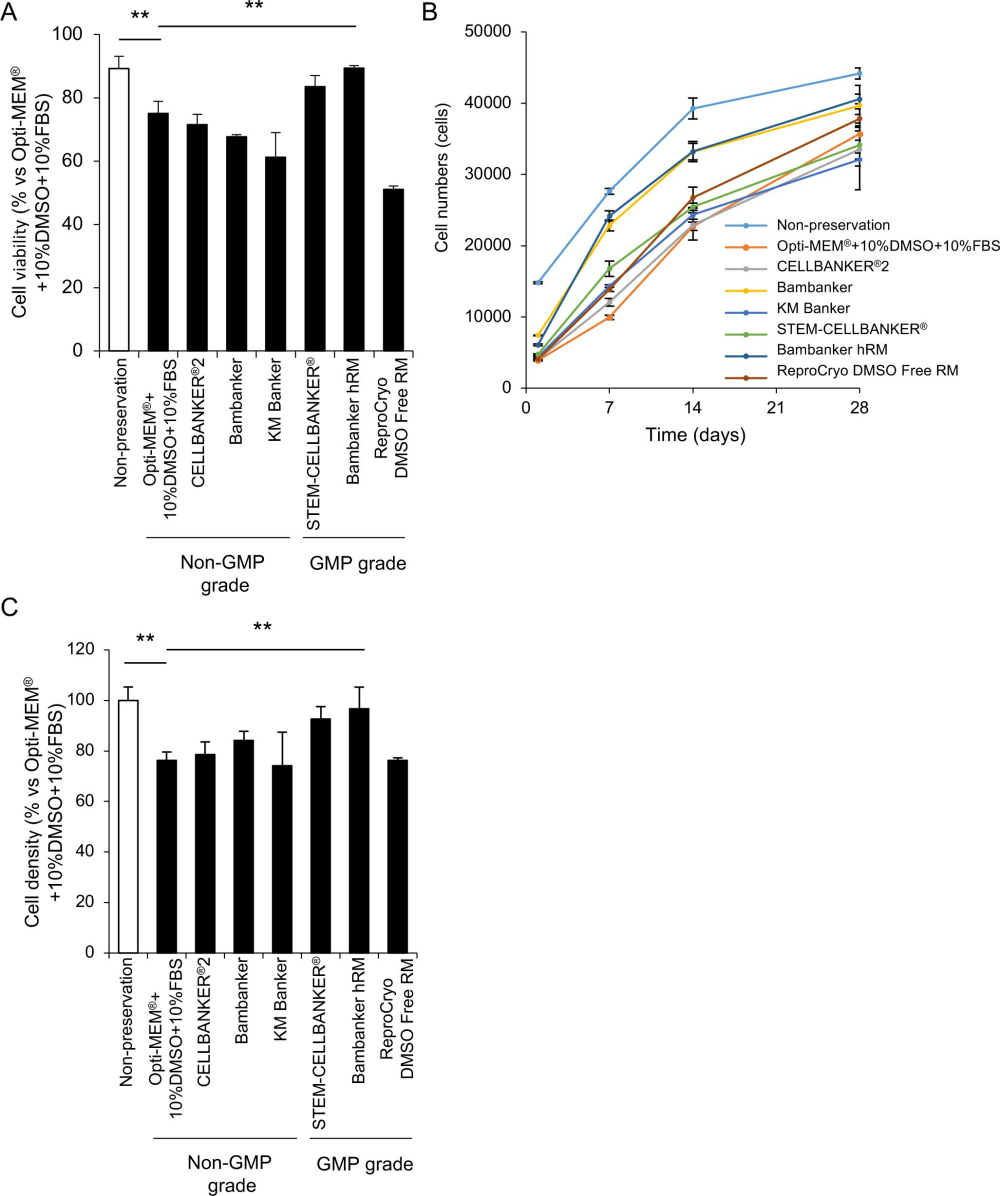
Screening of preservation reagents for cryopreservation. (A) Human corneal endothelial cells (HCECs) were cryopreserved for 14 days using various reagents. HCECs were resuspended in culture medium, and cell viability was determined by trypan blue staining. The percentage of viable HCECs was 75% using Opti-MEM + 10%DMSO + 10%FBS. The use of Bambanker hRM as a preservation reagent significantly increased the percentage of viable cells to 89.4%. Experiments were performed in triplicate. The statistical significance (vs. Opti-MEM + 10%DMSO + 10%FBS) was determined using Dunnett’s multiple-comparisons test (n = 3). **p<0.01. (B) HCECs were seeded at the cell density of 5.0×10^3^ in one well of a 96-well culture plate for 28 days after cryopreservation. All the preserved HCECs grew throughout the 28 days, but cells cryopreserved in Bambanker hRM and Bambanker tended to grow faster than HCECs preserved in other media. Experiments were performed in duplicate (n = 3). (C) HCECs were seeded at the cell density of 1.0×10^5^ in one well of a 24-well culture plate (5.2 × 10^4^ cells/cm^2^) for 28 days after cryopreservation. Cell density after cryopreservation and 28 days of culture was evaluated using ImageJ. The cell density after cryopreservation in Opti-MEM + 10%DMSO + 10%FBS decreased to 76.2% of the non-preserved control. The cell density after cryopreservation in Bambanker hRM was 96.6%, and significantly higher than that of the non-preserved control. Experiments were performed in triplicate. The statistical significance (vs. Opti-MEM + 10%DMSO + 10%FBS) was determined with Dunnett’s multiple-comparisons test (n = 3). **p<0.01.

We screened several preservation reagents for their effects on HCEC viability using HCECs at passage 5–10, a cell density of 1000–1500 cells/mm^2^ (due to the limited availability of cells), and the laminin-511 E8 fragment as a cell culture substrate. The percentage of viable cells was 89.2% for non-preserved cells harvested from the culture plate and immediately evaluated as a control, whereas the percentage of viable cells preserved with Opti-MEM + 10%DMSO + 10%FBS was 75.0%. The cells preserved with Bambanker hRM showed a significantly higher cell viability of 89.4% (Fig 2A).

HCECs were then cultured for 28 days after cryopreservation using the various reagents. All the cryopreserved HCECs grew throughout the 28 days, and all formed a contact-inhibited sheet like structure at approximately 21 to 28 days. Cells preserved with Bambanker hRM and Bambanker tended to grow faster than the other preserved HCECs (Fig 2B). HCECs preserved in Opti-MEM + 10%DMSO + 10%FBS and evaluated after 28 days of culture showed a decreased viability of 76.2% when compared to the non-cryopreserved control cells. By contrast, HCECs preserved in Bambanker hRM maintained a cell density of 96.6% of the non-preserved control (Fig 2C).

**Fig 3:**
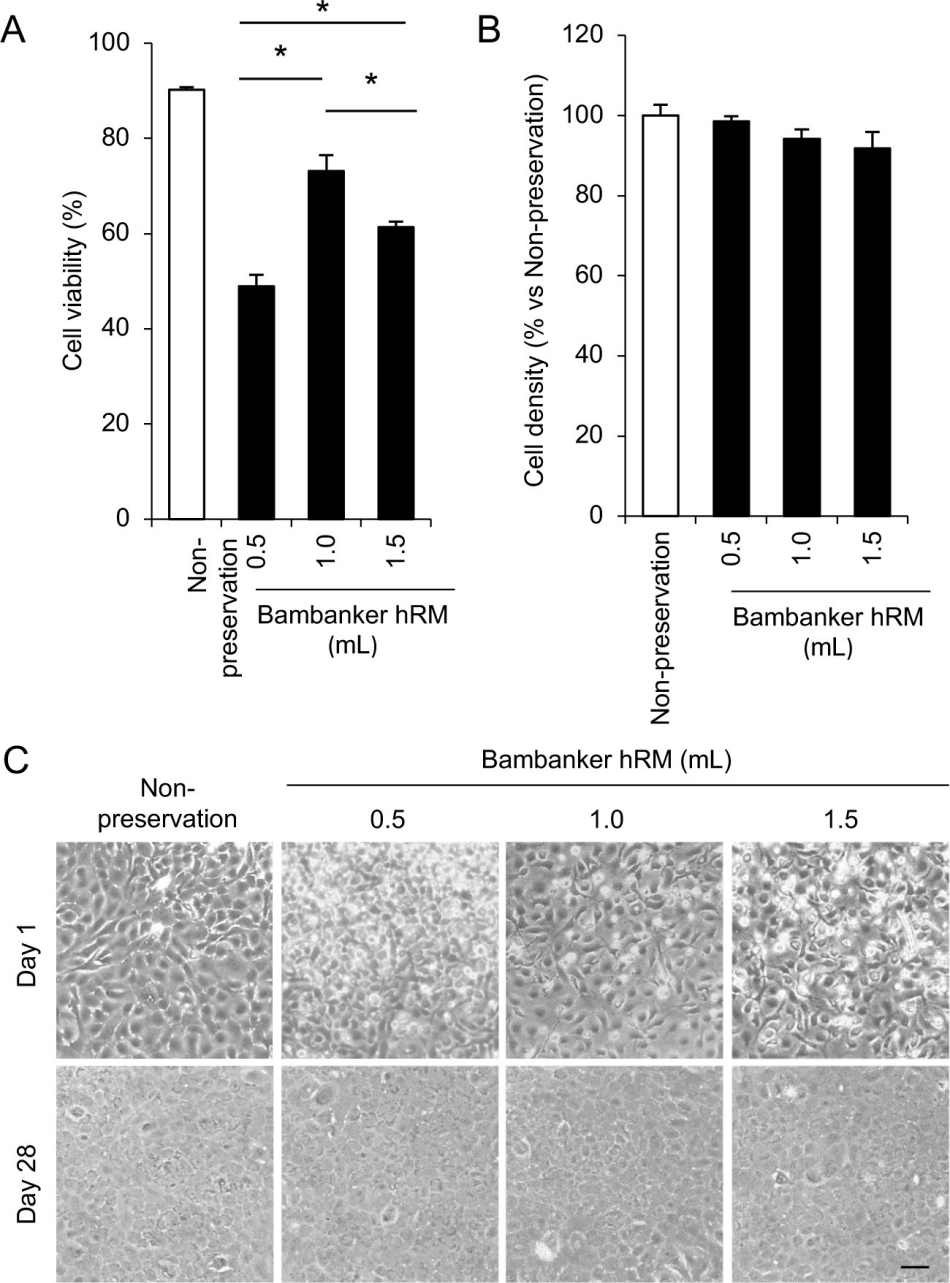
Optimization of cell concentration during cryopreservation. (A) A total of 5×10^5^ HCECs was suspended in 0.5, 1.0, or 1.5 ml Bambanker hRM, and cryopreserved for 14 days. The viability of HCECs recovered from frozen stock was determined by trypan blue staining. The percentage of viable HCECs was significantly higher when cells were suspended in 1.0ml Bambanker hRM than in 0.5 or 1.5ml. Experiments were performed in triplicate. The statistical significance (vs.1.0 ml) was determined with Dunnett’s multiple-comparisons test (n = 3). *p<0.05. (B) HCECs were seeded at the cell density of 1.0×10^5^ in one well of a 24-well culture plate (5.2 × 10^4^ cells/cm^2^) for 28 days after cryopreservation, and cell density was evaluated with ImageJ. Experiments were performed in triplicate (n = 3). (C) Representative phase contrast images showed that larger numbers of presumed dead cells were observed in HCECs cryopreserved by 0.5 and 1.5 ml Bambanker hRM in comparison to HCECs cryopreserved by 1.0 ml Bambanker hRM at day 1. HCECs preserved by 0.5, 1.0, and 1.5 ml Bambanker hRM formed hexagonal and monolayer sheet like structure at similar cell density at day 28. Scale bar: 200 μm.

We also evaluated the effect of the cell number per amount of Bambanker hRM on cell viability and cell density to optimize the preservation protocol. A total of 5×10^5^ HCECs were suspended in 0.5, 1, or 1.5 ml Bambanker hRM and cryopreserved for 14 days. The percentage of viable cells was significantly higher when 5×10^5^ cells were suspended in 1.0ml Bambanker hRM than in 0.5 or 1.5ml (Fig 3A). The cell density after 28 days of culture was similar when HCECs were suspended in 0.5, 1.0, and 1.5 ml Bambanker hRM (Fig 3B). Phase contrast images obtained at day 1 showed fewer cells attached to the culture plate and more presumably dead cells when HCECs were preserved with 0.5 and 1.5 ml Bambanker hRM than with 1.0 ml Bambanker hRM. At day 28, HCECs preserved with 0.5, 1.0, and 1.5 ml Bambanker hRM had a hexagonal shape and formed a monolayer sheet-like structure with a similar cell density (Fig 3C).

### Feasibility of use of Bambanker hRM to cryopreserve clinical grade HCECs

**Fig 4:**
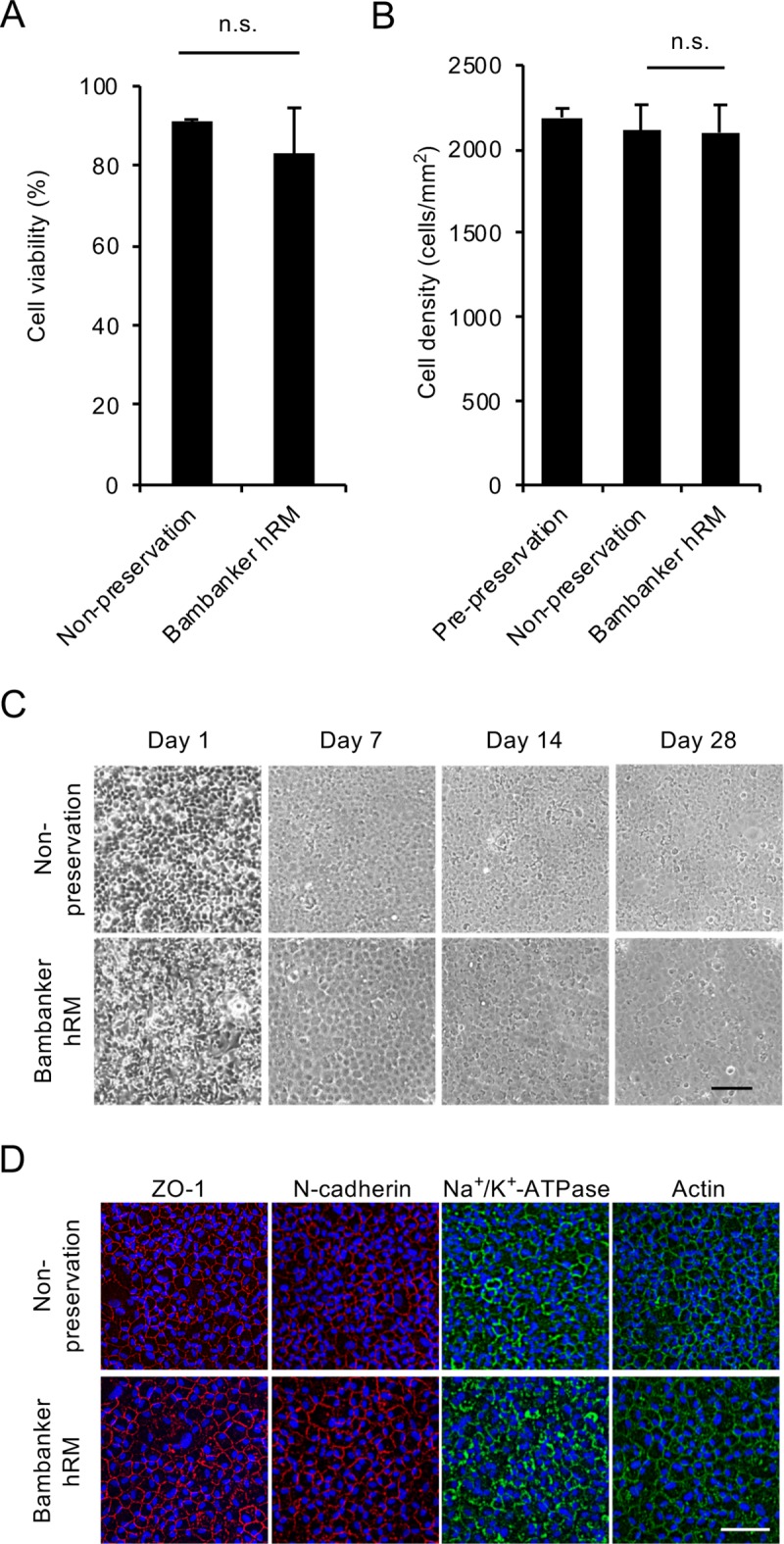
Feasibility of use of Bambanker hRM for preservation of clinical grade human corneal endothelial cells (HCECs). (A) HCECs similar in grade to those used clinically (passage 3–5; cell density greater than 2000 cells/mm^2^) were cryopreserved using Bambanker hRM. The percentage of viable cells was 83.2% after 14 days of cryopreservation and 91.0% for a non-preserved control. Experiments were performed in duplicate. (B) HCECs were cultured for 28 days after cryopreservation, and cell density was evaluated by ImageJ. Cell density was not significantly decreased by cryopreservation when compared to non-preserved control cells (2099 cells/mm^2^ and 2111 cells/mm^2^, respectively). The statistical significance (vs. non-preservation) was determined with Student’s t-test (n = 4). (C) Phase contrast images showed that HCECs preserved with Bambanker hRM exhibited similar cell growth to that of a non-preserved control. Scale bar: 200μm. (D) HCECs cultured for 28 days after cryopreservation were evaluated for function-related markers by immunofluorescence staining. Actin distribution and cell morphology were evaluated by actin staining. HCECs preserved with Bambanker hRM showed similar expression of ZO-1, N-cadherin, and Na^+^/K^+^-ATPase at the cell-cell border to that seen in the non-preserved control. Actin was distributed at the cell cortex in the preserved HCECs and showed the normal corneal endothelial pattern seen in the non-preserved control. Scale bar: 200 μm.

As a final test, we evaluated the feasibility of use of Bambanker hRM using HCECs at a grade typically used clinically for cell-based therapy (passage 3–5 and cell density greater than 2000 cells/mm^2^) [15]. The percentage of viable cells did not significantly decrease after cryopreservation when compared to the non-preserved control (83.2% and 91.0%, respectively) (Fig 4A). The cell density of the preserved HCECs at a confluent status reached a similar level to that of the non-preserved control after 28 days of cultivation (2099 cells/mm^2^ and 2111 cells/mm^2^, respectively) (Fig 4B). Phase contrast images showed that HCECs preserved with Bambanker hRM grew in a similar fashion to the non-preserved control and formed a cobblestone-like sheet structure (Fig 4C). Immunofluorescence staining showed that HCECs preserved with Bambanker hRM expressed ZO-1 (marker of tight junction), N-cadherin (marker of adherence junction), and Na^+^/K^+^-ATPase (marker of pump function) at the cell-cell border in a similar fashion to that observed in the non-preserved control. Actin staining showed that actin was distributed at the cell cortex, with no apparent fibroblastic changes, indicating a normal corneal endothelial pattern in both non-preserved and preserved HCECs (Fig 4D).

## Discussion

Cell culture of HCECs has been surprisingly challenging, because HCECs tend to go through fibroblastic changes, lose their corneal endothelial phenotype, show limited proliferative ability, and undergo senescence with cell density drop [17, 25–28]. These unfavorable features have severely restricted research in the field of corneal endothelium. For clinical purposes, HCECs need to be cultured in an efficient fashion while still maintaining their functional phenotype (i.e., the pump and barrier functions) and a sufficiently high cell density, which means that a more sophisticated protocol is required than is currently used for research purposes [29]. Consequently, the development of tissue engineering-based therapy for endothelial decompensation has been restricted by the difficulty in culturing HCECs, and many research groups, including ours, have devoted much effort to establish HCEC culture protocols [29]. For instance, we reported that the use of a Rho kinase inhibitor improved HCEC proliferation [27]. We also reported that the acquisition of a fibroblastic phenotype, which is suggested to be an example of the epithelial mesenchymal transition, is induced by activation of TGF-β signaling. Consequently, supplementation with a small-molecule TGF-β signaling inhibitor enables the culture of HCECs with the desired functional phenotype [28]. We also showed that the cell density drop was at least partially caused by cellular senescence due to activation of p38 MAPK signaling by culture stress and that the use of a p38 MAPK inhibitor and the laminin 511 E8 fragment as substrate could aid in overcoming this stress response [17, 24]. The culture methods vary depending on the research group, but multiple technical breakthroughs in the past decade have enabled the successful culture of HCECs [30]. Indeed, we have transplanted HCECs cultured according to our protocol into human patients with endothelial decompensation [15]. Our clinical outcome of restoration of corneal transparency without severe adverse effects indicates that current culture protocols may provide safe and functional cells.

In light of the fact that HCEC culture required technical breakthroughs, it is perhaps not surprising that cryopreservation of HCECs remains a challenge. Earlier study showed that HCECs maintained proliferative ability and phenotypic property after cryopreservation in freezing medium consisting 10% DMSO + 90% FBS [19]. More recently, Marquez-Curtis and colleagues demonstrated that slow cooling at 1°C/min in the presence of 5% dimethyl sulfoxide and 6% hydroxyethyl starch enabled the cryopreservation of HCECs, and these cryopreserved cells formed monolayer sheets [21, 23]. Though a vitrification procedure was reported using bovine CECs, the use of toxic cryoprotectants and the difficulty in controlling parameters, such as cooling and warming rates, are limitations of the protocol [31]. In our current study, we screened commercially available cryopreservation reagents and evaluated criteria that we are currently using in clinical settings, including cell density and expression of functionally related markers [15]. We showed that only half the HCECs were alive after cryopreservation in a mixture of basal culture medium (Opti-MEM + 10%DMSO + 10%FBS) and the cell density decreased to 76.2%. However, the present screening of cryopreservation agents identified Bambanker hRM as one that maintained cell viability after cryopreservation at a similar level to that obtained after immediate harvesting of non-preserved HCECs from a culture plate. Moreover, we showed that the cell density of clinical grade HCECs was not significantly decreased by cryopreservation with Bambanker hRM.

Bambanker hRM contains human serum albumin but not any xeno-derived materials. It was registered as a Master File by the PMDA (equivalent to the Drug Master Files of the FDA) in 2015 and is provided with Good Manufacturing Practice (GMP) compliance (https://www.n-genetics.com/products/search/detail.html?product_id=4551, accessed on July 8, 2018), suggesting that this reagent can be used in the clinical setting. One drawback of the present study is that all components included in Bambanker hRM are not disclosed. Therefore, the mechanisms by which Bambanker hRM suppress cell death during cryopreservation and thawing and maintain cell density remain unclear. Future research investigating the physiology of cell damage during cryopreservation and thawing of HCECs will be beneficial for further improvements in cryopreservation efficiency.

In conclusion, we have demonstrated that the use of Bambanker hRM enables the cryopreservation of HCECs by maintaining cell viability and cell density. Cryopreservation of HCECs has multiple advantages including the ability to accumulate stocks of master cells, to transport HCECs long distances, and to manufacture HCECs on demand from these stocks for use in cell-based therapy for treating endothelial decompensation.

## Supporting information

S1 File Raw data file. (XLSX)
